# The protein Deleted in Breast Cancer-1 (DBC1) regulates vascular response and formation of aortic dissection during Angiotensin II infusion

**DOI:** 10.1038/s41598-020-63841-8

**Published:** 2020-04-21

**Authors:** Laura Colman, Maria Caggiani, Alejandro Leyva, Mariana Bresque, Sally Liechocki, Clarissa M. Maya-Monteiro, Daniel Mazal, Carlos Batthyany, Aldo Calliari, Paola Contreras, Carlos Escande

**Affiliations:** 1 Laboratory of Metabolic Diseases and Aging, INDICyO Program, Institut Pasteur Montevideo, Montevideo, Uruguay; 2 Laboratory of Vascular Biology and Rational Drug Design, INDICyO Program, Institut Pasteur Montevideo, Montevideo, Uruguay; 3 Analytical Biochemistry and Proteomics Unit, Institut Pasteur Montevideo and Instituto de Investigaciones Biológicas Clemente Estable, Montevideo, Uruguay; 4 Departamento de Fisiología, Facultad de Medicina, Universidad de la República (UdelaR), Montevideo, Uruguay; 5 Departamento de Anatomía Patológica, Facultad de Medicina, Universidad de la República (UdelaR) and C.H Pereira Rossell, Montevideo, Uruguay; 6 Laboratório de Imunofarmacologia, Instituto Oswaldo Cruz, Fundação Oswaldo Cruz, Rio de Janeiro, Brazil; 7 Departamento de Biociencias, Facultad de Veterinaria, Universidad de la República (UdelaR), Montevideo, Uruguay

**Keywords:** Acute coronary syndromes, Hypertension

## Abstract

Cardiovascular diseases are among the main causes of morbimortality in the adult population. Among them, hypertension is a leading cause for stroke, heart disease and kidney failure. Also, as a result of arterial wall weakness, hypertension can lead to the development of dissecting aortic aneurysms, a rare but often fatal condition if not readily treated. In this work, we investigated the role of DBC1 in the regulation of vascular function in an ANGII-induced hypertension mouse model. We found that WT and DBC1 KO mice developed hypertension in response to ANGII infusion. However, DBC1 KO mice showed increased susceptibility to develop aortic dissections. The effect was accompanied by upregulation of vascular remodeling factors, including MMP9 and also VEGF. Consistent with this, we found decreased collagen deposition and elastic fiber fragmentation, suggesting that increased expression of MMPs in DBC1 KO mice weakens the arterial wall, promoting the formation of aortic dissections during treatment with ANGII. Finally, DBC1 KO mice had reduced cell proliferation in the intima-media layer in response to ANGII, paralleled with an impairment to increase wall thickness in response to hypertension. Furthermore, VSMC purified from DBC1 KO mice showed impaired capacity to leave quiescence, confirming the *in vivo* results. Altogether, our results show for the first time that DBC1 regulates vascular response and function during hypertension and protects against vascular injury. This work also brings novel insights into the molecular mechanisms of the development of aortic dissections.

## Introduction

Metabolic and aging diseases have become a main health concern worldwide. The increase in life expectancy, together with changes in lifestyle, mainly sedentarism, obesity and smoking have led to a dramatic increase in chronic non-infectious diseases, such as cardiovascular diseases (i.e. atherosclerosis, hypertension). Altogether, these diseases constitute the main causes of morbimortality worldwide^1^. Sustained hypertension can lead to heart attack and stroke, kidney failure, aortic dissections and aneurysms^1–3^. In this regard, Angiotensin-II (ANGII), one of the key mediators of the renin-angiotensin system (RAS), is known to play a crucial role in the development of hypertension. ANGII, acting mainly through AT1 receptor, promotes a variety of cellular responses, including hypertrophy, fibrosis, reactive oxygen species (ROS) production and tissue remodeling^4^.

Animal models have proved to be extremely useful for understanding the pathogenesis of hypertension. There are several animal models available to study vascular and renal hypertension and they all have their advantages and limitations in terms of recapitulating the human disease^5^. ANGII-mediated hypertension in mice by subcutaneous administration is by far the most widely used animal model for primary hypertension, accounting for nearly 50% of NIH-funded hypertension research^5^. This model has several distinctive advantages. First, it is possible to study the direct effect of ANGII on the vascular system. Second, it is useful to gain comprehensive insights into the molecular mechanisms of the RAS. Third, it is a very strong model in terms of reproducibility among species^5^. Also, animal models of ANGII-infusion are the best characterized and most widely used models of abdominal aortic aneurysms (AAA) or aortic dissections (AD)^6–12^.

In the vascular system, ANGII-mediated hypertension leads to remodeling of the arterial wall in order to cope with increased blood pressure. These changes involve, among others, extracellular matrix remodeling and increase in wall thickness. The latter is mainly caused due to proliferation and hypertrophy of vascular smooth muscle cells (VSMC)^13^. Alterations in normal vessel remodeling during hypertension can lead to abnormal and life-threatening responses. One of them is the generation of aortic dissection (AD), a condition in which patients are highly vulnerable to life-threatening complications and death, particularly when the ascending aorta is involved. In humans, AD pathogenesis is mostly associated with a tear of the arterial wall, where blood dissects in intima-media, creating a “false lumen”. Based on their localization, the Stanford classification differentiate between type A and B aortic dissections. A-type dissections occur in the ascending aorta, while B-type ones localize in the descending portion of the aorta. AD is often fatal within the first hours to days after the onset (acute stage). Alternatively, it may evolve to a subacute stage (months since onset) and then to a chronic stage. The diversion of blood flow into the false lumen can cause hemodynamic alterations leading to ischemia, malperfusion, aortic valve insufficiency and tamponade (compression of the heart). These can lead to sudden aortic rupture, circulatory failure and death in the majority of patients without timely treatment^3,14,15^.

It is important to notice that several lesions described previously as AAA, mainly in the model of ApoE-/- mice treated with ANGII, have been recently called into question as models of AD rather than AAA^11,12^. Despite reproducing several of the pathological features of human AAA such as elastin degradation, formation of intramural hematoma, aortic dissection, macrophage infiltration or thrombus formation, the ANGII-infused model of aneurism in mice seems triggered by different pathological mechanisms^11,12^. The most consistent lesion associated to ANGII-infusion models are the micro-ruptures of the vasa vasorum in the boundary of the tunica media with the adventitia, accompanied by a false channel formation with varying degrees of severity, and in some cases a lesion characterized by a dissected adventitia and an intramural hematoma (IMH). We therefore described these lesions as AD, a term that is currently used by other researchers^11,12^. Notably this pathogenic mechanism differs from the ones described for most of the AD seen in humans, where aortic dissection is characterized by development of an intimal flap caused by blood flowing into the media and forcing the intima and the adventitia apart (communicating AD). This intimal flap separates the true lumen (the normal pathway of blood flow in the aorta) from a false lumen. It is worth noting, bleeding of the vasa vasorum with formation of IMH may evolve into non-communicating AD in 30–40% of patients with IMH^16,17^.

Genetics, family history, age and gender, atherosclerosis and hypertension predispose to their development and progression^14^. Importantly, the molecular mechanisms that operate in the development of AD are still not completely elucidated. Several studies have shown that dysregulation of Matrix Metalloproteinases (MMPs), as well as angiogenic factors like VEGF^11^, contribute to the development of AD^18^. In particular, MMP-9 has been shown to play an important role in this condition^18^. Considering that mice models of ANGII recapitulate AD, also, MMP12 seems to be involved in the AD progression^9^. In addition, it has been reported that MMP-9 and MMP-2 knockout mice were resistant to develop such lesions^8^. However, it is unclear which are the molecular pathways controlling MMPs and angiogenic factors expression during hypertension and how they lead to the formation of AD.

Deleted in Breast Cancer 1 (DBC1; also known as CCAR2) is a nuclear protein that has many different functions in the regulation of metabolism^19^. DBC1 binds and regulates the biological activity of several transcription factors and epigenetic regulators, including SIRT1^20–22^, HDAC3^23^, p53^24^, BRCA1^25^, AR^26^, ER^27,28^, Rev-erb-alpha^29^ and PARP1^30^, among others.

Many lines of research support that DBC1 is involved in the control of metabolism and metabolic diseases^19,22,31–34^. We also showed that DBC1 KO mice have increased SIRT1 activity *in vivo* in liver and they are protected against non-alcoholic fatty liver disease^22^. In regards to cardiovascular diseases, we previously showed that DBC1 KO mice are protected against high-fat diet induced atherosclerosis^35^. However, our findings proved that protection against atherosclerosis was a consequence of increased lipid storage capacity in fat tissue rather than a local effect in blood vessels. Currently, there is no knowledge about the role of DBC1 in cardiovascular function.

In this work, we investigated the role of DBC1 in the regulation of vascular structure using a mouse model induced by ANGII infusion and hypertension. Both WT and DBC1 KO mice developed hypertension to a similar extent. However, we found a higher incidence of AD in DBC1 KO mice in response to ANGII infusion. Absence of DBC1 led to up-regulation of MMPs *in vivo* and *in vitro* in VSMC, including MMP9, which has been linked to the development of AD. These changes were accompanied by decreased collagen levels and elastin fibers fragmentation, suggesting that DBC1 regulates extracellular matrix dynamics during hypertension. Finally, we also found that DBC1 KO mice failed to augment wall thickness in response to ANGII treatment, which was accompanied by decreased VSMC proliferation *in vivo* and *in vitro*. Altogether, our results constitute the first *in vivo* evidence that DBC1 is implicated in the tissue remodeling in response to ANGII, and also brings novel insights into the molecular mechanisms that regulate the development and progression of aortic dissections.

## Materials and Methods

### General reagents and antibodies

All general reagents and chemicals were purchased from Sigma-Aldrich, including angiotensin II (ANGII, A9525), unless otherwise specified. Lipofectamine RNAiMax, Bradford protein assay reagent, Trizol and SuperScript II RT were bought from Invitrogen. SiRNAs oligos were purchased from Ambion (Negative Control 4390843; HDAC3 4390771) or Invitrogen (DBC1 MSS211964 and SIRT1 MSS234959). Antibodies were purchased from Bethyl (anti DBC1, 434 A), Abcam (anti tubulin 7291, anti BrdU 6326, anti KI67 16667), or Cell Signaling (anti Cyclin D1 9262, anti PCNA 92552). DNase I and Fast SYBR Green were purchased from Roche.

### Animal handling and experiments

All mice used in this study were maintained at the Institut Pasteur de Montevideo Animal facility (UATE). The experimental protocol was approved by the Institutional Animal Care and Use Committee of the Institut Pasteur de Montevideo (CEUA, Protocol number 014–14). All the studies described were performed according to the methods approved in the protocol and following all international guidelines and legal regulations. WT and whole-body DBC1 KO mice were in a C57BL6/J pure background. DBC1 KO mice were backcrossed into C57BL/6 J for more than 10 generations in order to ensure genetic purity. Mice received standard chow and water *ad libitum*.

### Angiotensin II treatment in mice

Alzet mini-osmotic pumps (model 2004, 28 days delivery) were subcutaneously implanted in mice under isoflurane anesthesia. Prior to the implant, pumps were filled either with ANGII (1 mg/kg/day) or vehicle (NaCl 0.9%) and left for 7 and 28 days. All the results are representative of at least 3 independent experiments. Each group contained at least 5 mice per group. A total of 101 mice were infused with either ANGII or vehicle (50 WT and 51 DBC1 KO).

### Blood pressure measurements

Blood pressure (BP) in mice was measured by a non-invasive CODA system (HT 4, version 1.06; CODA PCS40 software, version 4.1; Kent Scientific), using a Volume Pressure Recording (VPR) sensor. This methodology consists of a cuff placed on the tail to occlude the blood flow. Upon deflation, the VPR sensor uses a specially designed differential pressure transducer to measure the blood volume in the tail. Before recording, animals were allowed to acclimate (1 h at 30 °C). BP was registered in the afternoon, twice a week for two consecutive days during 6 weeks (2 weeks before the pumps implantation and during the 4 weeks of treatment). BP values for each week correspond to the mean value of both days (15 recordings per session). When Systolic BP values were obtained with a tail blood volume below 15 μl they were discarded.

### Metabolic cages

Mice were placed in individual metabolic cages for 48 h at the fourth week of treatment. The first day was for acclimatization and the following 24 h were the ones considered for the analysis of urine and water intake. Urine production was measured and centrifuged and the supernatant was frozen for subsequent procedures.

### ***In vivo*Bromodeoxyuridine (BrdU) incorporation**

5 Bromo-2′-deoxyuridine (BrdU) was injected into mice (100 mg/kg, IP, single dose) at the first, second and third week of treatment. At the first or fourth week, mice were sacrificed and a segment of descending thoracic aorta was fixed in 4% paraformaldehyde (PFA) for 24 h for immunohistochemistry.

### BrdU Immunohistochemistry

Aortic sections were obtained from paraffin-embedded tissue and BrdU immunolabel were performed according to standard procedure. The paraffin sections (5 µM) were deparaffinized and rehydrated, incubated with 2% H_2_O_2_ for 20 minutes to block non-specific peroxidase signals. Briefly, samples were incubated with 2 M HCl for 15 minutes at 37 °C, blocked with 2% BSA-0.03% Triton x-100 during 1 h, incubated with BrdU antibody and revealed using an anti-rat -HRP-diaminobenzidine. Hematoxylin was used as counterstaining.

### Tissue isolation and AD diagnosis

Mice were anesthetized with intraperitoneal ketamine and xylazine (180 and 24 mg/kg, respectively). Depth of anesthesia was assessed by toe-pinch procedure and absence of muscular tone. Aortas were isolated after the first or fourth week of ANGII infusion, and either frozen in liquid nitrogen for subsequent molecular biology methods or fixed in 4% PFA. The presence of AD was evidenced *in situ* by macroscopic analysis of the whole aorta (ascending and descending). Once identified, AD was diagnosed under stereoscopic microscopy, as a blood clot surrounded by greatly expanded adventitial tissue and neovasculature on the outer surface, that made the artery difficult to remove. In all cases, the nature of the lesion was confirmed by histological analysis. Aorta scheme is illustrated to show different portions used for analysis (Supplementary methods). A portion of thoracic aorta was used to immunohistochemistry and staining techniques: Hematoxylin & Eosin (H&E) and Verhoeff (VF). In the cases when AD was observed macroscopically, tissue was processed to histological analysis stained with H&E and VF. Finally, a section of abdominal aorta below AD was used for molecular biology processing.

### Cell culture

Vascular smooth muscle cells (VSMCs) were obtained by outgrowth from abdominal aorta explants from WT or DBC1 KO male mice as previously described by others^36^. VSMCs were cultured in full medium containing DMEM supplemented with 10% fetal bovine serum (FBS), 2 mmol/L glutamine, 100 U/mL penicillin, 100 mg/mL streptomycin. Cells were cultured in a water-jacketed incubator at 37 °C and 5% CO_2_.

### Transfection procedure

For siRNA experiments, cells were plated in six well plates in medium used for VSMCs. When cultures reached 80% confluence, cells were transfected with 30 nM siRNA oligos (non-targeting negative control, DBC1, HDAC3 and SIRT1 using 25 pmol Lipofectamine RNAiMax. After 24 h post transfection this procedure was repeated. Cells were harvested 72 h after the first transfection.

### Western Blotting

Cells or tissues (50–100 mg tissue) were lysed using NETN buffer (25 mM Tris pH 8.0, 100 mM NaCl, 1 mM EDTA, 0.5% NP40) or RIPA buffer (25 mM Tris pH 8.0, 150 mM NaCl, 1% NP-40, 0.1% SDS), respectively. Protein concentrations were determined using the Bradford protein assay reagent. Proteins were separated in SDS-PAGE gels and transferred to polyvinylidene fluoride (PVDF) membranes, which were then blocked in Tris-buffered saline containing 0.2% Tween 20 and 5% nonfat milk. Membranes were then incubated overnight with anti DBC1, Cyclin D1, PCNA, P27 or tubulin. After that, secondary antibodies were incubated 1 h, washed and detected using chemiluminescence. Densitometry of bands was analyzed using Image J.

### Histological Evaluation of tissues

Paraffin sections (5 µm) were stained with H&E, Picrosirius red (PIC) for collagen and Verhoeff (VF) for elastic fibers stain. The specimens were observed under a light microscope (40×, 200×, 400X magnification). In order to measure the wall thickness of the aortic rings, we used a custom designed plugin for Image J. The procedure starts by clicking in the center of the aorta lumen. Then, 4 lines passing at that point are drawn, marking the aorta wall at 8 different places. Those are the 8 places where the wall is measured by clicking at the intima and at the outer limit of the media without considering the adventitia layer. The wall thickness is automatically obtained as the average of those 8 measurements. Collagen deposition was evaluated in intima-media aortic ring using the Wake Segmentation plugin from Image J, reporting results as area (%) occupied by collagen.

### Cell proliferation

VSMCs were counted, plated in 10 cm plate and cultured to 60% confluence. After that, FBS was removed for 48 h to induce cellular quiescence. Later, FBS was restored and cells collected at different time points. Total viable cells were selected by propidium iodide exclusion and counted by flow cytometry using BD Accuri C6 cytometer. Cell proliferation state under described experimental conditions was further confirmed by quantification of Ki67-positive cells, using immunofluorescence.

### ANGII Treatment of VSMC

Cells were grown until 90% confluence and then incubated with 0.1% FBS for 48 h. After that, cells were treated with 100 nm ANGII (or vehicle) for 12 h as previously described by others^37^.

### RNA Isolation and qPCR

Cells and tissues, were homogenized in Trizol for RNA extraction according to manufacturer’s protocol. DNase I treatment was used to eliminate genomic DNA contamination. Reverse transcription was done using SuperScript II RT and quantitative reverse transcription polymerase chain reaction (qRT-PCR) was performed using Fast SYBR Green Mix. Relative quantification of changes in gene expression were expressed in relation to a housekeeping gene. Expression was calculated as fold increase with respect to control condition. Primers containing the following sequences were synthesized by Integrated DNA Technology (IDT) and listed in Table 1.

**Table 1:** List of primers used for qPCR.

**Gene target**	**Forward (5′to 3′)**	**Reverse (5′to 3′)**
DBC1	CTGTGCCAACAGAAAGCCAC	GAGACAGGTTGACACAGCGA
Metalloproteinase 2	ACCTGAACACTTTCTATGGCTG	CTTCCGCATGGTCTCGATG
Metalloproteinase 9	GCAGAGGCATACTTGTACCG	TGATGTTATGATGGTCCCACTTG
Metalloproteinase 12	GCTGTCACAACAGTGGGAGA	ATGCTCCTGGGATAGTGTGG
VEGF-A	CAGGCTGCTGTAACGATGAA	TTTGACCCTTTCCCTTTCCT
β-Actin	AGCCATGTACGTAGCCATCC	GCTGTGGTGGTGAAGCTGTA
SIRT1	ATGACGCTGTGGCAGATTGTT	CCGCAAGGCGAGCATAGAT
HDAC3	ATGCCTTCAACGTGGGTGAT	AGAAGCCAGAGGCCTCAAAT

### Statistical analysis

Results are expressed as mean ± SEM unless otherwise specified. Shapiro Wilk Analysis were performed to confirm normal distributions. Unpaired t-test was used to compare two independent groups. In multiple comparisons, analysis of variance (ANOVA) followed by Bonferroni’s post hoc test, *P* < *0.05* was considered to be significant. Mann-Whitney U test was used to compare two independent samples (groups) in cases where sample distribution was not normal. For comparisons of proportions, Fisher’s exact test was used.

## Results

### ANGII promotes hypertension to a similar extent in WT and DBC1 KO mice

**Figure 1:**
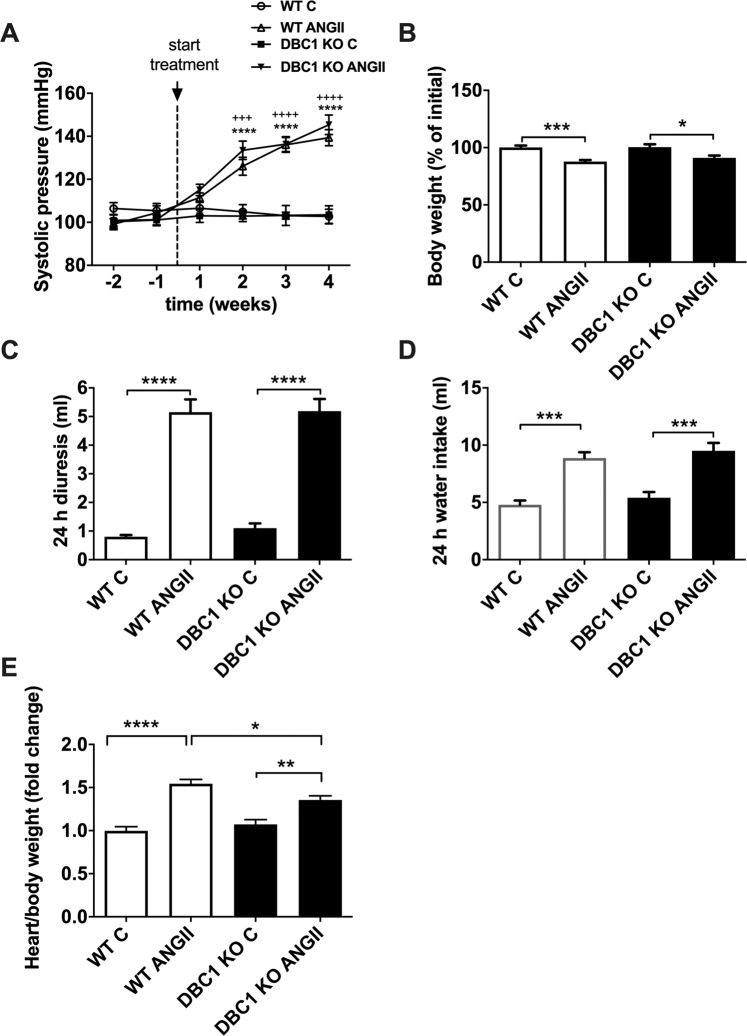
ANGII induces arterial hypertension in WT and DBC1 KO mice (**A**) Systolic blood pressure measured in WT and DBC1 KO mice treated with 1 mg/kg/day ANGII infusion or vehicle (C, control group). (**B)** Body weight measured in WT and DBC1 KO mice treated with ANGII or vehicle (C, control group). Mice treated with ANGII lost weight during the treatment while their littermate controls treated with vehicle did not. Diuresis (**C)** and Water intake (**D)** measured after 4 weeks treatment increased in WT and DBC1 KO mice treated with ANGII. (**E)** Hearts weights from WT and DBC1 KO treated with ANGII were measured after treatment. WT and DBC1 KO hearts treated with ANGII were heavier than those from control mice. Two-way ANOVA with Bonferroni’s post hoc test were done for comparisons between ANGII and control groups in (**A)**.Three or 4 symbols at week comparisons mean *P* < 0.001 or 0.0001, respectively (* for WT and + for DBC1 KO). In (**B–E)** *, **, *** and **** mean *P* < 0.05, 0.01, 0.001 and 0.0001, respectively. One-way ANOVA with Bonferroni’s post hoc test were done for comparisons between experimental groups (n = 7–10).

In order to investigate the role of DBC1 on vascular function, we used the model of ANGII-induced vascular hypertension. WT and DBC1 KO mice were exposed to subcutaneous infusion of ANGII (1 mg/kg/day) for 4 weeks. Under these conditions, we found that both WT and DBC1 KO mice developed hypertension in a similar manner (140 mm Hg, measured by systolic blood pressure, Fig. 1A). We also measured body weight (Fig. 1B), diuresis (Fig. 1C), and water intake (Fig. 1D). In all groups ANGII had a significant effect in all these parameters and there was no difference between WT and DBC1 KO mice. ANGII treatment also induced cardiac hypertrophy both in WT and DBC1 KO mice; however, DBC1 KO mice were partially protected against hypertrophy (Fig. 1E).

### DBC1 KO mice show increased incidence of aortic dissections (AD) in response to ANGII infusion

**Figure 2:**
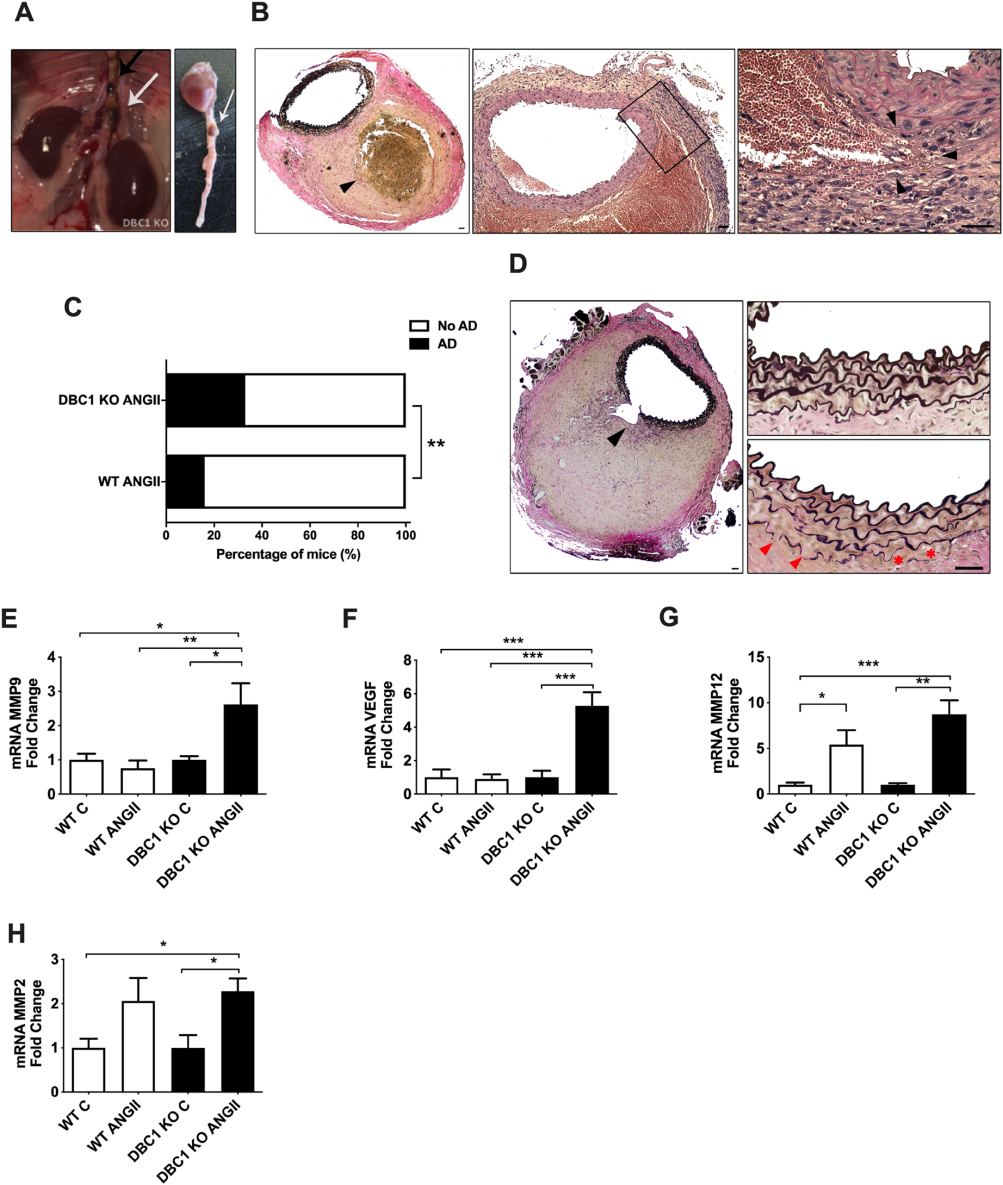
Absence of DBC1 increases the incidence of aortic dissection (AD) in response to ANGII. A-B) Macroscopic and histological characterization of AD. (**A)** *In situ* descending aorta and isolated heart with aorta. Arrows show examples of AD. A case of a secondary thoracic AD identified in DBC1 KO mouse is also shown (black arrow, left panel). (**B)** Histological analysis of intra mural hematoma (IMH) leading to an AD. Left, panoramic representative of IMH. Verhoeff staining of the cross section of the aorta is shown. Black arrowhead indicates the thrombus. Center and right, H&E staining of the cross section of the aorta is shown. Right image shows a higher magnification of the center image, highlighting the dissected adventitia and an intramural space filled with blood. Note the presence of intact erythrocytes at the periphery of the hematoma. Arrowheads indicate a point to tearing of the intima media. Scale bar: 40 µm. (**C)** Incidence of AD in WT and DBC1 KO mice treated with ANG II (n = 37 and 36, WT and DBC1 KO mice, respectively). Fisher’s exact test was used to compare groups. (**D)** Representative Verhoeff staining of AD. Black and red arrowheads indicate intima media wall disruption (plausible a late effect consequence of IMH expansion) and ruptured elastic fibers, respectively. Scale bar: 40 and 500 µm, left and right panel, respectively. (**E–H)** mRNA expression of MMP9, VEGF, MMP12 and MMP2 in WT and DBC1 KO mice aortas after 4 weeks of treatment with ANGII or vehicle (**C **). One-way ANOVA with Bonferroni’s post hoc test for multiple comparisons were done. *, ** and *** means *P* < 0.05, 0.01 and 0.001, respectively (n = 8 per group).

When we performed post-mortem analysis of vascular injury in aortas, we found that the DBC1 KO mice showed increased susceptibility to the development of AD (Fig. 2A–D and Supplementary Fig. 1). The presence of AD evidenced after ANGII treatment was detected by macroscopic analysis in the descending aorta (Fig. 2A). In most cases, AD developed in the abdominal portion of the aorta, close to the renal arteries (Fig. 2A, left, white arrow). In some rare cases, a second lesion was observed in the descending thoracic aorta (Fig. 2A, left, black arrow). No lesions were detected in the ascending portion of the aorta. Histological analysis of the tissue confirmed the nature of the lesions. Consistent with previous reports, we found evidence of micro-ruptures in the boundary of the tunica media with the adventitia, such as presence of extravasated erythrocytes (Supplementary Fig. 2), occurring mostly in close proximity to the orifice of side arterial branches of abdominal aorta. More important, collections of extravasated red cells, suggestive of a false channel formation were also present. In most severe cases we observed a lesion characterized by a dissected adventitia and an intramural space filled with coagulated blood, without apparent increase of the aortic lumen diameter (Fig. 2B). In some cases, the margins of the thrombus penetrated into the adventitia and periaortic adipose tissue. As the IMH start to remodel, the space become filled of fibrin with only a small layer of still intact erythrocytes at the periphery. In more older lesions, no red blood cells were visible as they were replaced by spindle cells, granulation tissue and collagen deposition. To our knowledge, these pathological findings are compatible with virtually all existing literature on the abdominal lesions of ANG II-infused ApoE -/- mice. When the mice were examined after 4 weeks of ANGII treatment, WT mice showed a scarce incidence of such lesions (15% of the mice treated with ANGII). However, nearly 40% of the DBC1 KO mice developed AD in response to ANGII infusion (Fig. 2C). Interestingly, DBC1 KO mice showed higher incidence of AD as early as 1 week after the onset of ANGII infusion (Supplementary Fig. 1), when blood pressure was beginning to rise (Fig. 1A). When ANGII dose was lowered from 1 mg/kg/day to 0.6 mg/kg/day, WT mice did not develop AD, but DBC1 KO mice still had around 20% of AD incidence (Supplementary Fig. 1). No spontaneous ADs were detected either in WT or DBC1 KO mice in control conditions.

In some cases a partial intima-media layer break that resulted in marked dilation of the lumen was observed. This was accompanied by an interruption in the continuity of the elastic fibers (Fig. 2D, left panel). Also, we observed intermediate stages of extracellular matrix re arrangements characterized by weakening and partial breakdown of elastic fibers localized mainly in close proximity to intramural hematoma (arrowheads, Fig. 2D, right panel).

It is well known that alteration of the dynamics of the extracellular matrix, including changes in the expression of several matrix MMPs as well as pro-angiogenic factors are involved in the development of AD^8–10,38,39^. Moreover, MMP9 a protein that has been shown to degrade elastic fibers^40^, and proposed biomarker of AD in patients^18^ was differentially up-regulated in the aortas from DBC1 KO mice treated with ANGII (Fig. 2E). This difference was also seen when mice were treated with ANGII for only one week (Supplementary Fig. 1). In order to rule out that elevation of MMP9 expression in DBC1 KO mice was a consequence of AD rather than a causative effect, we analyzed its expression in those treated with ANGII that did not develop AD. In these mice, MMP9 expression was increased in DBC1 KO mice compared to WT, although for both genotypes there was a further increase when AD was present (Supplementary Fig. 2). Furthermore, when we analyzed MMP9 expression in the descending thoracic aorta, we also found increased expression of MMP9 in DBC1 KO mice treated with ANGII (Supplementary Fig. 2). Also, VEGF, a pro-angiogenic factor that has been linked to the development of AD (reported originally as AAA)^41^ was also significantly increased in the aortas from DBC1 KO mice treated with ANGII compared to WT mice (Fig. 2F). The expression of MMP12 and MMP2 was also increased in response to ANGII, but there was no significant difference between WT and DBC1 KO mice (Fig. 2G,H, respectively).

### **Down-regulation of DBC1 in vascular smooth muscle cells*in vitro*recapitulates the*in vivo*effects**

**Figure 3:**
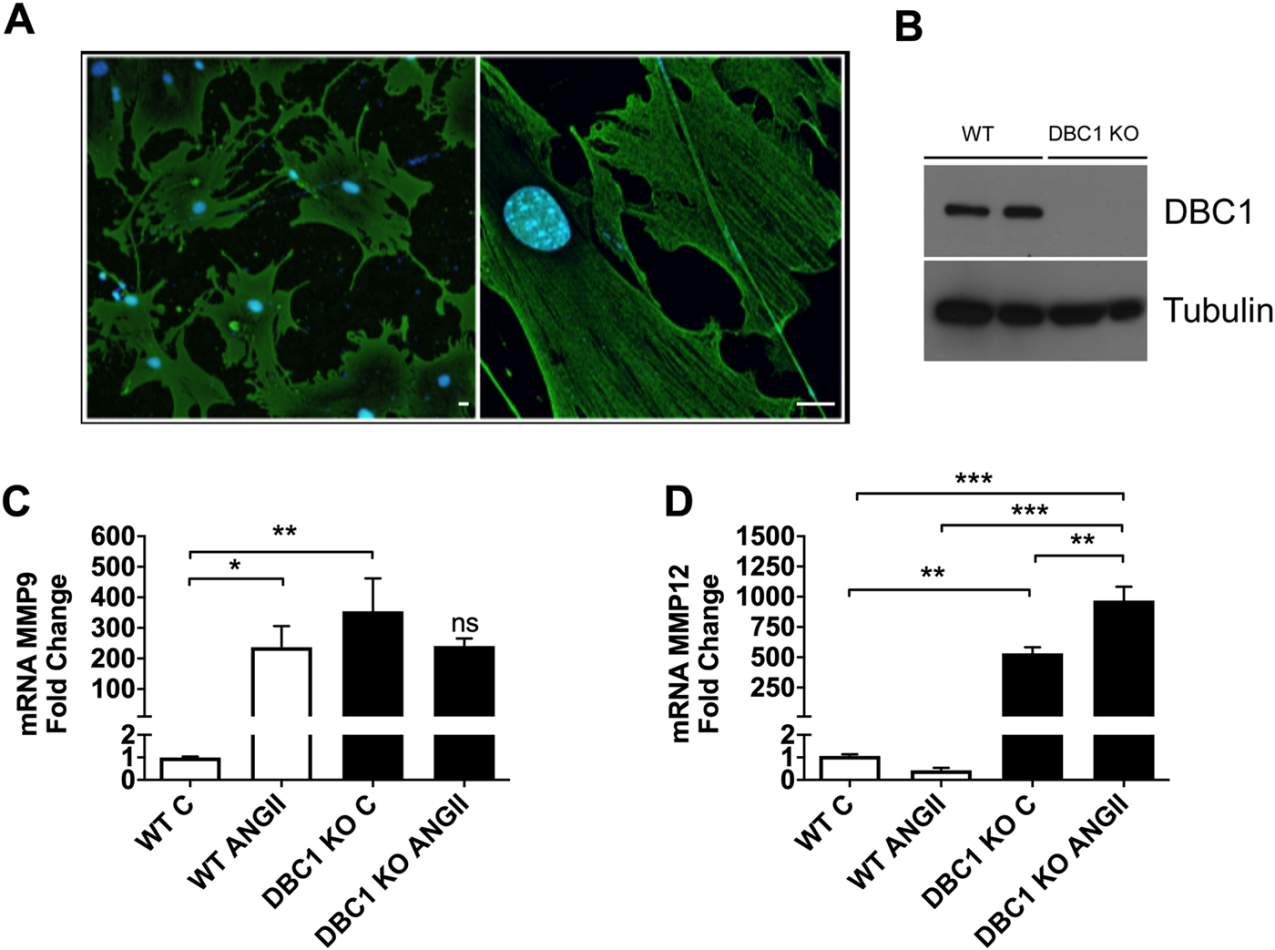
Downregulation of DBC1 in Vascular Smooth Muscle Cells (VSMC), increases the expression of aortic dissection (AD) markers. (**A**) Immunofluorescence image of VSMC purified and cultured from abdominal aorta using alpha-actin antibody (green), marker of VSMC. Left, panoramic view (100×) showing purity of the culture. Right, high magnification (630×) showing alpha-actin filaments in VSMC. Scale bar: 15 µm. (**B)** Representative western blot for DBC1 expression in proliferating VSMC purified from WT and DBC1 KO mice. (**C,D)** mRNA expression of MMP9 and MMP12 in WT and DBC1 KO VSMC, treated with vehicle (C) or 100 nM ANGII for 12 h. The results are shown as the mean ± SEM from at least 3 independent experiments. One-way ANOVA with Bonferroni’s post hoc test for multiple comparisons was done. *,** and *** means *P* < 0.05, 0.01 and 0.001, respectively.

In order to determine if changes in the expression of MMPs and angiogenic factors were a direct consequence of DBC1 down-regulation, we purified and cultured abdominal VSMC from WT and DBC1 KO mice. Purity of cells was confirmed by alpha-actin expression (Fig. 3A). DBC1 expression in WT VSMC was confirmed by western blot (Fig. 3B). Similar to what happened *in vivo*, we found that cells purified from DBC1 KO mice had increased expression of MMP9, MMP2 and MMP12 (Supplementary Fig. 3). Also, treatment of WT cells with ANGII *in vitro* promoted an increase in MMP9 expression to a similar extent of DBC1 KO cells in basal conditions. In DBC1 KO cells ANGII treatment did not further increase MMP9 expression, suggesting that in these cells MMP9 expression was constitutively up-regulated (Fig. 3C). Moreover, in the case of MMP12, ANGII promoted a further increase in DBC1 KO cells (Fig. 3D). To further confirm our results, we knocked-down DBC1 by siRNA in WT VSMC (Supplementary Fig. 3). We found that knock-down of DBC1 promoted the up-regulation of VEGF, MMP9, MMP12, and MMP2, further supporting the results obtained *in vivo* and *in vitro* comparing WT and DBC1 KO. We also analyzed if SIRT1 or HDAC3, two of the main targets of DBC1^22,23^ were involved in this regulation. Neither SIRT1 nor HDAC3 downregulation by siRNA affected the DBC1-dependent response on MMPs expression, suggesting that DBC1 is being targeted through a different molecular pathway (Supplementary Fig. 3).

### **DBC1 KO mice show decreased collagen deposition and impaired arterial wall thickening in response to ANGII treatment**

**Figure 4:**
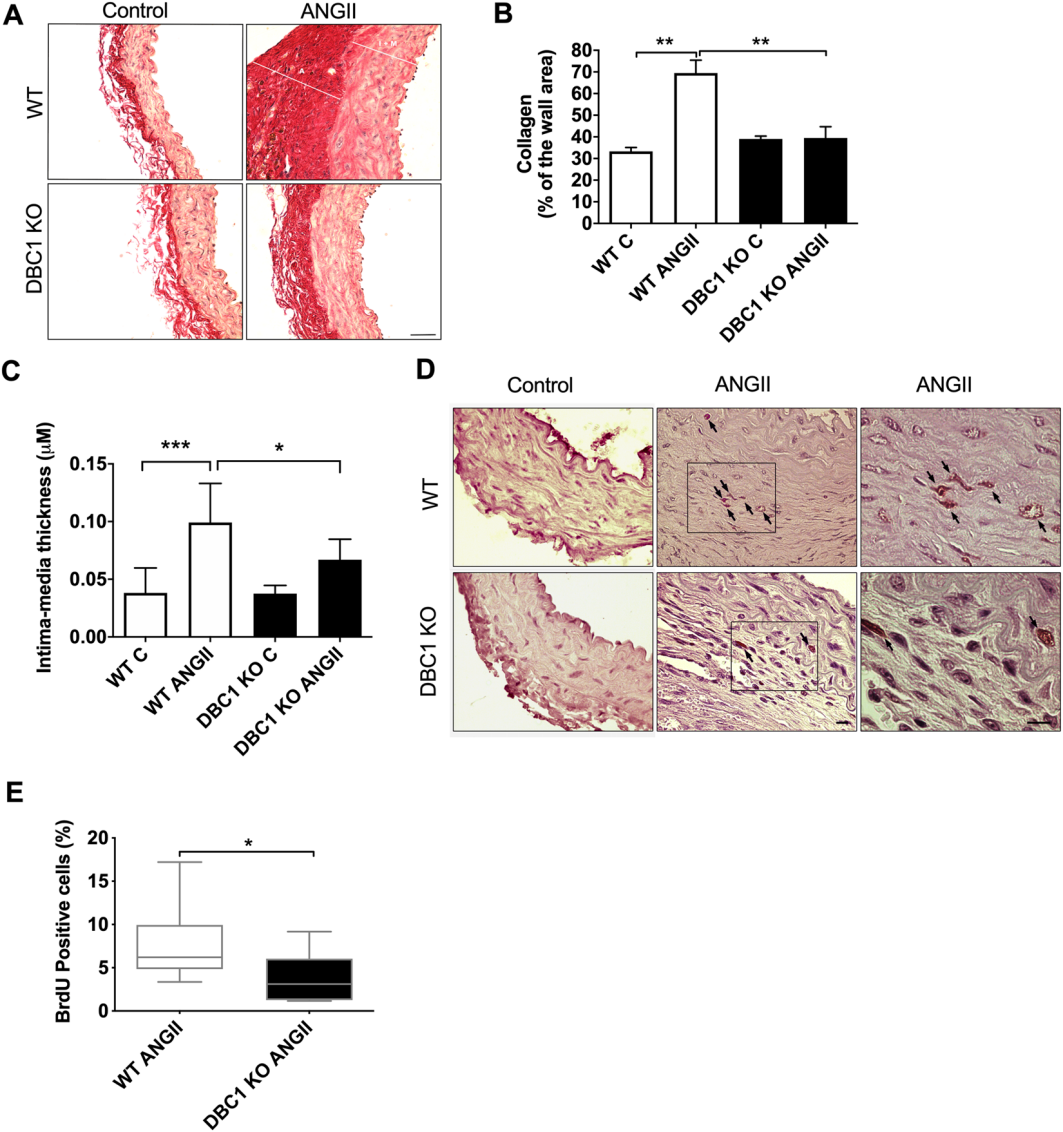
DBC1 KO mice show a failed collagen turnover and impaired vascular proliferation in response to ANGII-induced hypertension. (**A**) Representative images of aorta stained with Picrosirius red from Control or ANGII-treated WT and DBC1 KO mice. Collagen fibers are stained in red. White lines indicate adventitia (**A**) and intima-media (I + M). Scale bar: 40 µm. (**B)** Collagen fibers quantitation from the intima-media tunica (n = 3 for control mice and 4 for ANGII treated mice). One-way ANOVA with Bonferroni’s post hoc test for multiple comparisons was done. ** mean *P* < 0.01. (**C**) Quantitation of the intima-media tunica thickness in WT and DBC1 KO mice in Control and ANGII-treated conditions. One-way ANOVA with Bonferroni’s post hoc test for multiple comparisons was done. * and *** means *P* < 0.05, and 0.001, respectively. (**D**) Representative pictures of Bromodeoxyuridine (BrdU) incorporation in WT and DBC1 KO mice aortas in Control and ANGII-treated conditions. Arrows indicate BrdU positive cells (DAB staining). Slides were counterstained with Hematoxylin. Scale bar: 20 µm. (**E**) Quantitation of BrdU positive cells in the intima-media tunica of WT and DBC1 KO mice treated with ANGII. One-tailed Mann-Whitney U test was done (DBC1 KO median of frequency = 2.8%; WT median of frequency = 6.2%), U = 7. No positive signals were detected in control mice (n = 7 per group).* means *P* < 0.05.

MMP9 degrades not only elastic fibers but also collagen fibers in the arterial wall^40^. Since decreased collagen deposition has also been linked to weakening of the arterial wall and increased susceptibility to AD^18^, we also measured collagen content in the arterial wall in WT and DBC1 KO mice. Consistent with the increased MMP9 expression, we found decreased collagen deposition in the intima-media layer in the DBC1 KO mice in response to ANGII (Fig. 4A,B and Supplementary Fig. 1). Consistently, we also observed a clear difference in the thickness of the intima media between WT and DBC1 KO mice after the treatment with ANGII (Fig. 4C). One of the main responses to hypertension is the cellular proliferation of the intima-media cells, mainly VSMC, with a consequent thickening of the arterial wall^42–45^. Interestingly, it has been shown that in patients with AD, the thickness of the intima-media layer is reduced compared to control subjects^14,46^ and that VSMC proliferation capacity is involved in the pathogenesis of aortic aneurysms and AD^46,47^. Recently, we showed that deletion of DBC1 delays cell cycle progression in previously quiescent cells, both *in vitro* and *in vivo*^48^. In order to evaluate VSMC proliferation capacity, we measured BrdU incorporation after 1 week of ANGII treatment where it has been shown that VSMC can already be proliferating during hypertension^49^ and also when AD incidence was already higher in DBC1 KO mice (Supplementary Fig. 1). We found that proliferation was not different between WT and DBC1 KO mice at that time, which was coincidental with no changes in wall thickness among genotypes (Supplementary Fig. 1). However, when we measured BrdU incorporation later (at weeks 2–3), we found that DBC1 KO mice showed significantly less BrdU positive cells in the intima-media layer than WT mice (Fig. 4D–E). This suggests that failure to regain cell cycle in quiescent VSMC contributes to artery wall weakness during hypertension, although it may not be directly involved in the development of AD.

### **Vascular smooth muscle cells from DBC1 KO mice fail to proliferate*in vitro***

**Figure 5:**
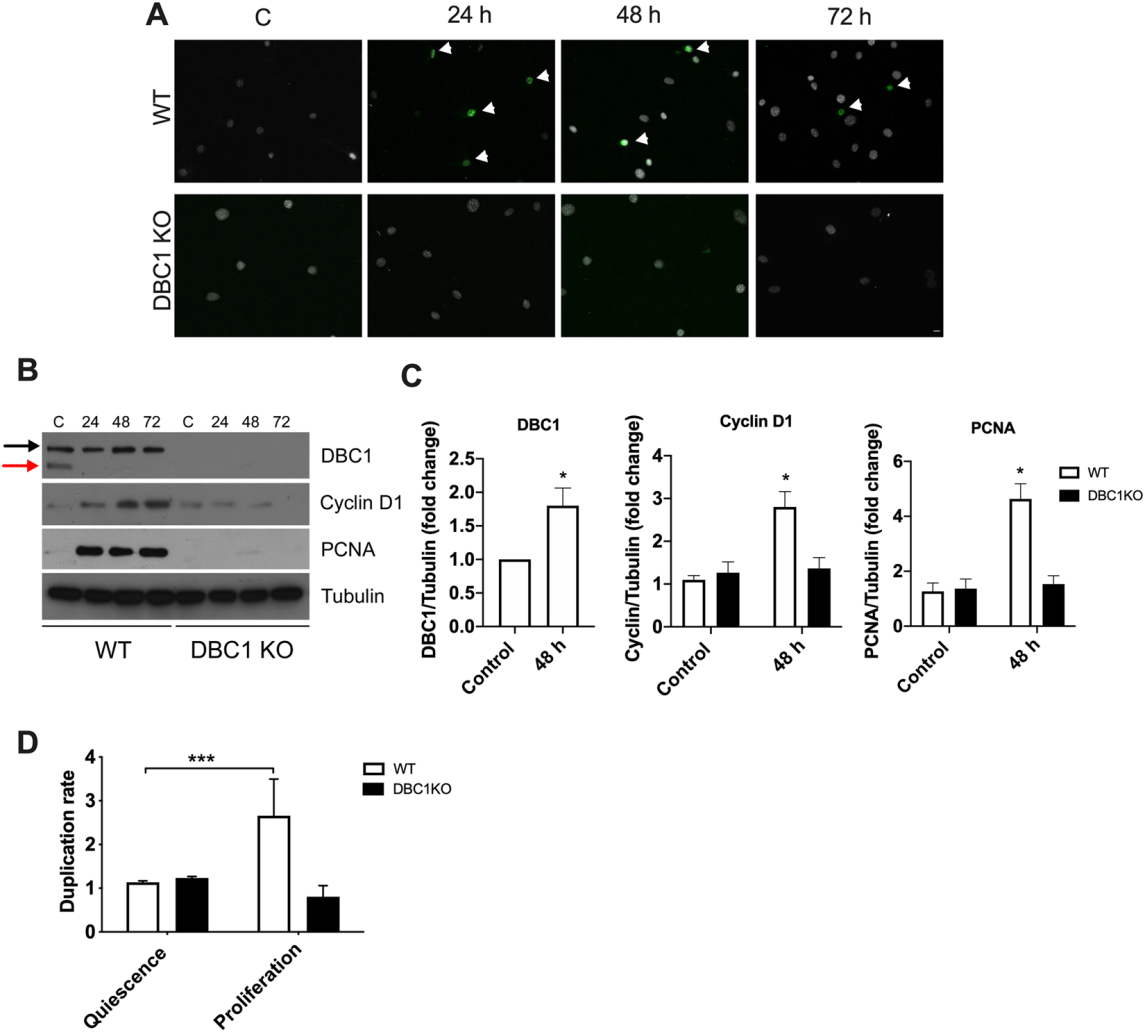
Vascular smooth muscle cells from DBC1 KO mice fail to proliferate *in vitro*. VSMC from WT and DBC1 KO mice aortas were grown and synchronized to a quiescent state by 48 h of serum deprivation. After that, serum was restored and cell cycle progression followed for 24, 48 and 72 h. (**A)** Ki67 staining in WT and DBC1 KO cells. Arrowheads point to Ki67 positive cells (green). DAPI was used as marker of total cell numbers (white). Scale bar: 15 µm. (**B)** Representative western blot of cell cycle markers expression (Cyclin D1 and PCNA) in WT and DBC1 KO VSMC at quiescent state after 48 h of serum withdrawal (**C**), and at different time points after serum replenishment (24, 48 and 72 h). Black arrow shows DBC1 and red arrow shows DN-DBC1. (**C)** Quantitation of DBC1 protein expression in quiescent state (Control) and after 48 h of serum replenishment (48 h). Results shown were from 3 independent experiments. (**D)** Quantitation of the replication rate for cell number in quiescent and proliferative, post-quiescent states (72 h) of WT and DBC1 KO VSMC. Cells were grown in complete media (DMEM + 10% FBS for proliferation), later incubated with DMEM with no serum for 48 h(quiescence). After 48 h of serum deprivation, FBS was restored and cells collected 72 h later. Propidium Iodide (PI) negative cells were selected and counted at the different conditions. Three independent experiments were used for quantitation. One-way ANOVA with Bonferroni’s post hoc test for multiple comparisons were done. * means p < 0.05.

As mentioned above, we recently showed that deletion of DBC1 delays cell cycle progression in cells that leave quiescence, both *in vitro* using Mouse Embryonic Fibroblasts (MEFs) and *in vivo* in a model of liver regeneration^48^. In order to evaluate if failure to leave quiescence could explain decreased *in vivo* proliferation of VSMC, we cultured VSMC form WT and DBC1 KO mice and measured cell cycle progression. WT and DBC1 KO VSMC were synchronized into quiescence by serum deprivation^48^ and later allowed to re-enter cell cycle by restoring normal serum. Serum restoration in WT cells was accompanied by up-regulation of the proliferation marker Ki67 (Fig. 5A). However, DBC1 KO cells showed no positive cells, even after 72 h of incubation (Fig. 5A). Also, serum deprivation of WT VSMC led to the appearance of DN-DBC1, a novel form of DBC1 present in quiescent cells^48^ (Fig. 5B). DN-DBC1 was rapidly downregulated after serum restoration. This was accompanied by a time-dependent increase of Cyclin D1 and PCNA expression, markers of cell cycle progression (Fig. 5B,C). On the other hand, VSMC cultured from DBC1 KO mice failed to up-regulate any of these cell cycle progression markers (Fig. 5B,C). We measured cell number under the same conditions by flow cytometry and found that while WT VSMC duplicated its population 72 h after serum restoration, DBC1 KO VSMC completely failed to proliferate (Fig. 5D), supporting the results obtained *in vivo*.

## Discussion

In this study we show for the first time, that DBC1 is directly involved in the regulation of vascular function. Moreover, we found that deletion of DBC1 increases the incidence of aortic dissections (AD) triggered by ANGII. Mechanistically, our results show that absence of DBC1 increases the expression of vascular remodeling factors, including MMPs, specially MMP9, that are involved in the development of AD both in mice models and humans^2,3,8,9,18,38,41^. Also, we found that DBC1 KO mice have impaired arterial wall thickening response to ANGII. This is coincident with the fact that DBC1 KO vascular smooth muscle cells failed to leave their quiescent state and re-enter the cell cycle, both *in vitro* and *in vivo*.

We and others have extensively shown that DBC1 plays major roles in metabolism and metabolic diseases^19,22,31–35^. DBC1 regulates SIRT1 activity in the liver and adipose tissue during different metabolic states^22,31^. Furthermore, we also showed that DBC1 KO mice are protected against vascular injury and atherosclerosis, due to increased fat adipose tissue buffer capacity during obesity^35^. In this work we found that ANGII promotes hypertension to a similar extent in WT and DBC1 KO mice, although the latter were partially protected against cardiac hypertrophy. This result is consistent with several reports which have shown that SIRT1 activation protects against cardiac hypertrophy^50,51^. To our surprise, we found that DBC1 KO mice had a higher incidence of AD triggered by the treatment. Our results suggest that DBC1 affects different aspects of vascular response during hypertension, which are discussed below.

### **Deletion of DBC1 increases the expression of matrix remodeling factors that correlate with the incidence of AD in mice and humans**

Our results show that DBC1 KO mice exhibit increased incidence of AD in response to ANGII. This effect was as early as 1 week after ANGII infusion (Supplementary Fig. 1), when blood pressure was beginning to rise and hypertension was still to be established. Interestingly, when ANGII dose was decreased from 1 mg/kg/day to 0.6 mg/kg/day ADs disappeared from WT mice. However, DBC1 KO mice still showed incidence of AD, although to a lower extent (Supplementary Fig. 1), supporting the notion that absence of DBC1 predisposes to develop vascular lesions. The role of hypertension in the development of AD in the ANGII-infusion model is not completely clear. Reports have shown that AD promoted by ANGII are sensitive to telmisartan and irbesartan^52^. However, others have suggested that ANGII-induced AD develop even in the absence of hypertension^53^. The fact that AD can develop before the onset of hypertension (1 week of ANGII infusion) and also that in DBC1 KO mice they still develop at lower doses of ANGII, suggest that AD and hypertension may be considered as independent events. However, further studies will be needed in order to determine if AD formation in DBC1 KO is independent from blood pressure.

Gene expression analysis of aortas after ANGII infusion showed that DBC1 KO mice had increased expression of MMP9 and VEGF compared to WT mice. Both proteins are up-regulated in mice models of AD, and also in aortic lesions analyzed from patients^3,54,55^. In fact, MMP9 KO mice are protected against aortic lesions^10^. Also, inhibition of VEGF protects against the development of aneurysmatic lesions in mice^38,39,41,55^. Increased expression of MMP9 and VEGF in DBC1 KO mice aortas was paralleled by decreased collagen deposition in the intima-media of the aorta, suggesting differential collagen turnover in the DBC1 KO mice, a factor that has also been linked to the formation of AD^40^. *In vitro* analysis of cultured VSMC showed that DBC1 is a direct regulator of the expression of MMP9 and VEGF, but also MMP2 and MMP12, suggesting that DBC1 is a general regulator of matrix remodeling factors. These results differ from a previous report using NCI-N87 and MKN-45 gastric cancer cell lines, where authors showed that DBC1 *knock-down* promotes the downregulation of MMP9 and MMP2^56^. Our findings, together with our previously published research, suggests that DBC1 might play different and even opposite roles in transformed and normal cells^48^.

It is important to notice that only DBC1 KO mice showed a clear up-regulation of MMP9 mRNA after one week of ANGII treatment (Supplementary Fig. 1C). In fact, MMP9 was up-regulated in ANGII-treated DBC1 KO aortas even in mice that did not develop AD (Supplementary Fig. 2C). Although it is not possible to establish a clear causative effect between MMP9 expression and the development of AD in the DBC1 KO mice, altogether our results point in that direction. There was an intriguing difference between what we observed in tissues and the findings in VSMC. *In vitro*, there were clear differences in MMPs expression between genotypes under basal (no ANGII stimulation) conditions, while this was not detected *in vivo* in untreated WT and DBC1 KO mice. We cannot provide a definite explanation for this discrepancy. One possibility is that the expression of MMPs by VSMC *in vivo* is too low to detect differences among genotypes. Another possibility is that other cell types rather than VSMC are the main driving force for the observed phenotype. In this regard, it has been shown that macrophages^57^ and neutrophils^58^ that infiltrate vascular tissue during hypertension are an important source of MMPs.

Also, although *in vitro* analysis in VSMC showed that other MMPs besides MMP9 and VEGF are up-regulated in response to DBC1 deletion, we cannot establish for sure if this is also true *in vivo*. It is plausible that those changes, especially in the case of VEGF, are secondary to the vascular lesion rather than a causative phenomenon. All the issues mentioned above need to be clearly established in order to fully understand how DBC1 is regulating vascular response to hypertension.

Many of the biological effects of DBC1 are mediated by regulation of SIRT1 activity, and in fact it was recently shown that SIRT1 activity protects against AD in the context of caloric restriction (CR). The authors showed that specific knock-down of SIRT1 in VSMC *in vivo* abolished the protective effect of CR against AD^7^. We evaluated if either SIRT1 or HDAC3, another target of DBC1^23^, could be mediating the DBC1 effect of vascular remodeling factors expression, and we found that neither SIRT1 nor HDAC3 seem to be involved in this phenomenon (See Supplementary Information).

As it was mentioned in the introduction, DBC1 has many molecular targets, which makes it very laborious to single out a molecular effector of its function. In this case, based on the available data, we can propose two possible DBC1-dependent pathways implicated in the development of AD. The first one involves the regulation of the p53 protein. It has been recently shown that loss of p53 sensitizes to ANGII-induced AD during caloric restriction^59^. Indeed, p53 regulates VSMC function^60^. Since DBC1 is a direct regulator of p53^24^, it is possible that the DBC1-p53 axis is involved in our results. Another possible downstream effector of DBC1 in AD may be the long non-coding RNA (lncRNA) MALAT1. This lncRNA was recently involved in the regulation of aneurysmatic lesions by regulating VSMC function^61^. MALAT1 also regulates MMPs expression and cell invasiveness in cancer cells^62,63^. MALAT1 is directly regulated by DBC1^64^, suggesting that dysregulation of this lncRNA could explain the DBC1-dependent effect on AD in response to ANGII. Which of these pathways, or any other still to be known, are involved in the DBC1-dependent AD incidence needs to be further investigated.

### **Absence of DBC1 impairs vascular cell proliferation response triggered by hypertension**

Besides showing altered expression of vascular remodeling factors, the cells belonging to the intima and media layers from DBC1 KO mice showed impaired proliferation *in vivo* in response to ANGII treatment. This was evidenced by a decreased rate of BrdU incorporation into DNA and paralleled by a failure to increase the thickness of tunica-media in response to hypertension. Several reports have shown that VSMC proliferation capacity is compromised in AD^14,46,47^ and that failure of VSMC could be a cause for aortic wall weakening and aneurysm formation. Recently, we showed that deletion of DBC1 delays cell proliferation in previously quiescent cells, both *in vitro* and *in vivo* during liver regeneration^48^. In fact, when DBC1 KO VSMC were made quiescent by serum deprivation, they failed to re-enter cell cycle upon serum restoration. This was evidenced both by the expression cell cycle progression markers and by cell count. Quiescent VSMC from WT mice showed the appearance of the putative quiescence marker DN-DBC1^48^. Serum restoration triggered the down-regulation of DN-DBC1 and up-regulation of cell cycle progression markers. DBC1 KO VSMC were unable to up-regulate these proteins and failed to progress through cell cycle. These results suggest that, similar to the liver, the ratio between DBC1 and DN-DBC1 plays a role in the control of cell cycle progression of VSMC. Thus, this may affect the response of VSMC to vascular injury *in vivo*. Interestingly, it has been shown that SIRT1 overactivation in VSMC inhibits proliferation after vascular injury^65^. We propose that SIRT1 overactivation might impair the proliferation of VSMC in DBC1 KO mice in response to ANGII. Also, we speculate that under basal conditions the expression of DN-DBC1, which does not bind to SIRT1^48^, contributes to maintaining the quiescent state of VSMC by increasing SIRT1 activity. Upon vascular injury, DN-DBC1 might be fully replaced by DBC1, promoting cell proliferation in intima-media thickening.

**Figure 6:**
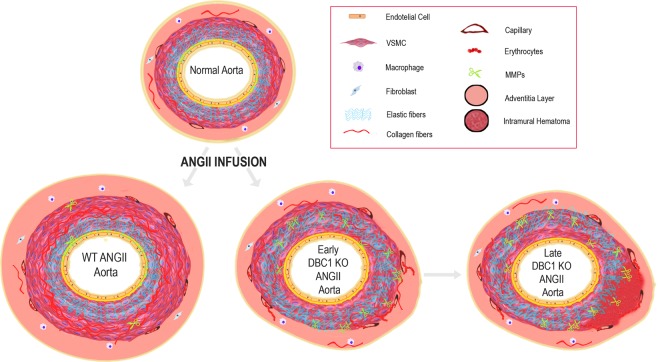
Working model for DBC1 role in the control of vascular response during ANGII-mediated hypertension. In basal conditions, the aorta is composed of a heterogeneous mixture of smooth muscle, nerves, endothelial cells, fibroblast-like cells, and a complex extracellular matrix which maintains balance to guarantee an appropriate blood flow. During hypertension, ANGII promotes VSMCs proliferation, extracellular matrix turnover, and inflammatory environment. However, in aortas from DBC1 KO mice, increased MMPs expression (represented as small scissors) leads to arterial wall weakening. This effect, in combination with micro ruptures of small vessels in the vasa vasorum and tunica media, leads to the formation of IMH and AD. Also, decreased VSMC proliferation in response to hypertension in DBC1 KO mice exacerbates the impaired vascular response to hypertension.

Based on all the results presented here, we generated a working model for DBC1 role in the control of vascular response during ANGII-mediated hypertension (Fig. 6). We propose that during hypertension, absence of DBC1 leads to increased expression of vascular remodeling factors, including MMPs and VEG. As a consequence, there is an increase in collagen and elastin turnover, thus weakening the arterial wall. The weakening of the arterial wall, in combination with micro-ruptures of small vessels that nurture the vasa vasorum and tunica media leads to the formation of an IMH and later aortic dissection. In addition, the lack of a proper proliferative response by DBC1 KO cells might further contribute to the ongoing aortic wall degeneration process.

## Conclusions

This work constitutes the evidence that DBC1 is directly involved in the control of vascular response to injury by regulating the expression of vascular remodeling factors and also the proliferative response of VSMC. Furthermore, it strengthens the emerging role of DBC1 as a key regulator of non-transformed cells and their transition from quiescent to proliferative states. Finally, our findings could contribute to the understanding of the molecular mechanisms that underlie the development of AD.

## Supplementary information

Supplementary Information.
