# Supplementary material for: The protein Deleted in Breast Cancer-1 (DBC1) regulates vascular response and formation of aortic dissection during Angiotensin II infusion
Source: Sci Rep. 2020 Apr 21;10:6772. doi: 10.1038/s41598-020-63841-8 (PMC7174338; doi:10.1038/s41598-020-63841-8)

The protein Deleted in Breast Cancer-1 (DBC1) regulates vascular response and formation of aortic dissection during Angiotensin II infusion

Laura Colman (1), Maria Caggiani (1,4), Alejandro Leyva (2,3), Mariana Bresque (1), Sally Liechocki (5), Clarissa M. Maya-Monteiro (5), Daniel Mazal (6), Carlos Batthyany (2), Aldo Calliari (1,7) Paola Contreras (1,4) and Carlos Escande (1)*

Supplementary Information

Supplementary methods

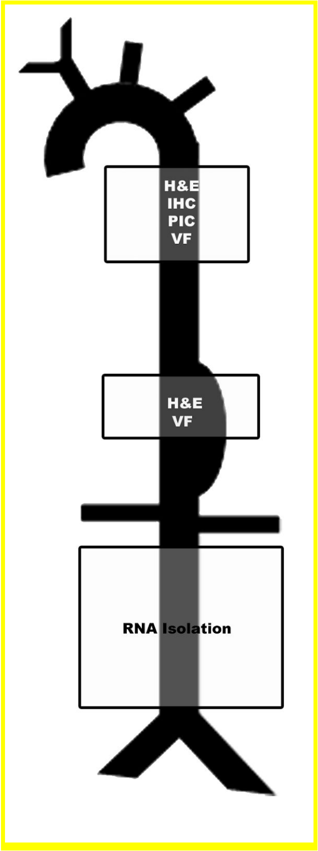

Schematic diagram of aorta tissue processing in mice.

Supplementary Results

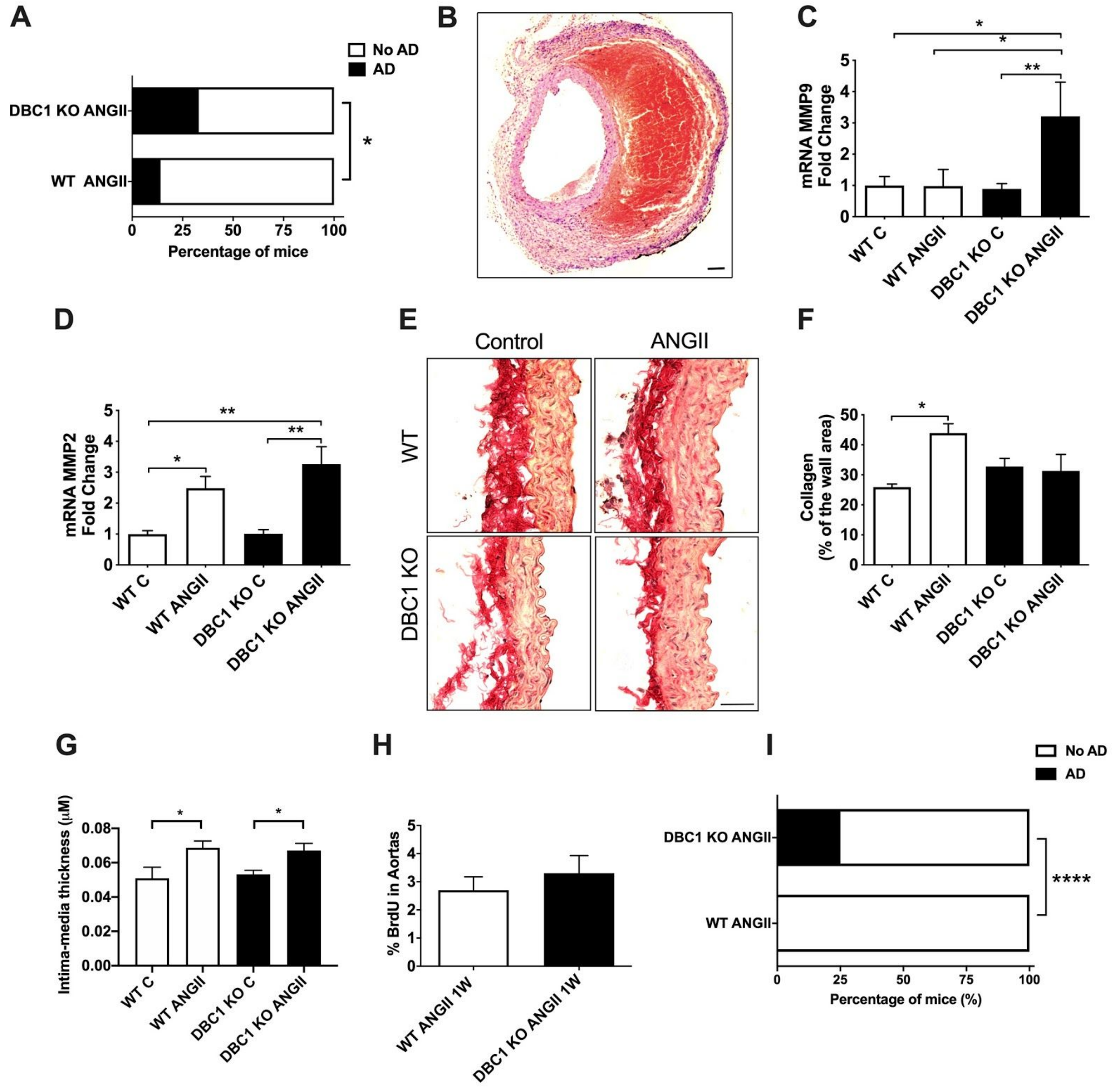

Supplementary figure 1. A) Incidence of AD in WT and DBC1 KO mice treated with ANG II for 1 week (n= 7 and 8, WT and DBC1 KO mice, respectively). Fischer´s exact test was used to compare groups. **B)** H&E staining of the cross section of the aorta at the site of the AD was shown. Scale bar: 40μm. **C-D)** mRNA expression of MMP9 and MMP2 in WT and DBC1 KO mice aortas in either control (C) or after 1 week of treatment with ANGII. **E)** Representative images of aortas stained with Picrosirius red

from control or ANGII-treated WT and DBC1 KO mice. Collagen fibers are stained in red. Scale bar: 40μm. **F)** Collagen fibers quantitation from the intima-media tunica (n= 3 for control mice and for ANGII treated mice). **G)** Quantitation of the intima-media tunica thickness in WT and DBC1 KO mice in control and ANGII-treated conditions. **H)** Quantitation of BrdU positive cells in the intima-media tunica of WT and DBC1 KO mice treated with ANGII (n= 4 and 3, WT and DBC1 KO mice, respectively). No positive signals were detected in control mice (n=7 per group). One-tailed Mann-Whitney U test was used, $P > 0.05$. **I)** Incidence of AD in WT and DBC1 KO mice treated with a lower ANGII dosis (0.6 mg/kg/day), for 4 weeks. No spontaneous AD were detected neither in WT or DBC1 KO mice in control conditions. Fischer´s exact test was used to compare groups. (C,D,F and G) One-way ANOVA with Bonferroni's post hoc test for multiple comparisons was done. *, ** and *** means $P < 0.05$, 0.01 and 0.001, respectively. (H)

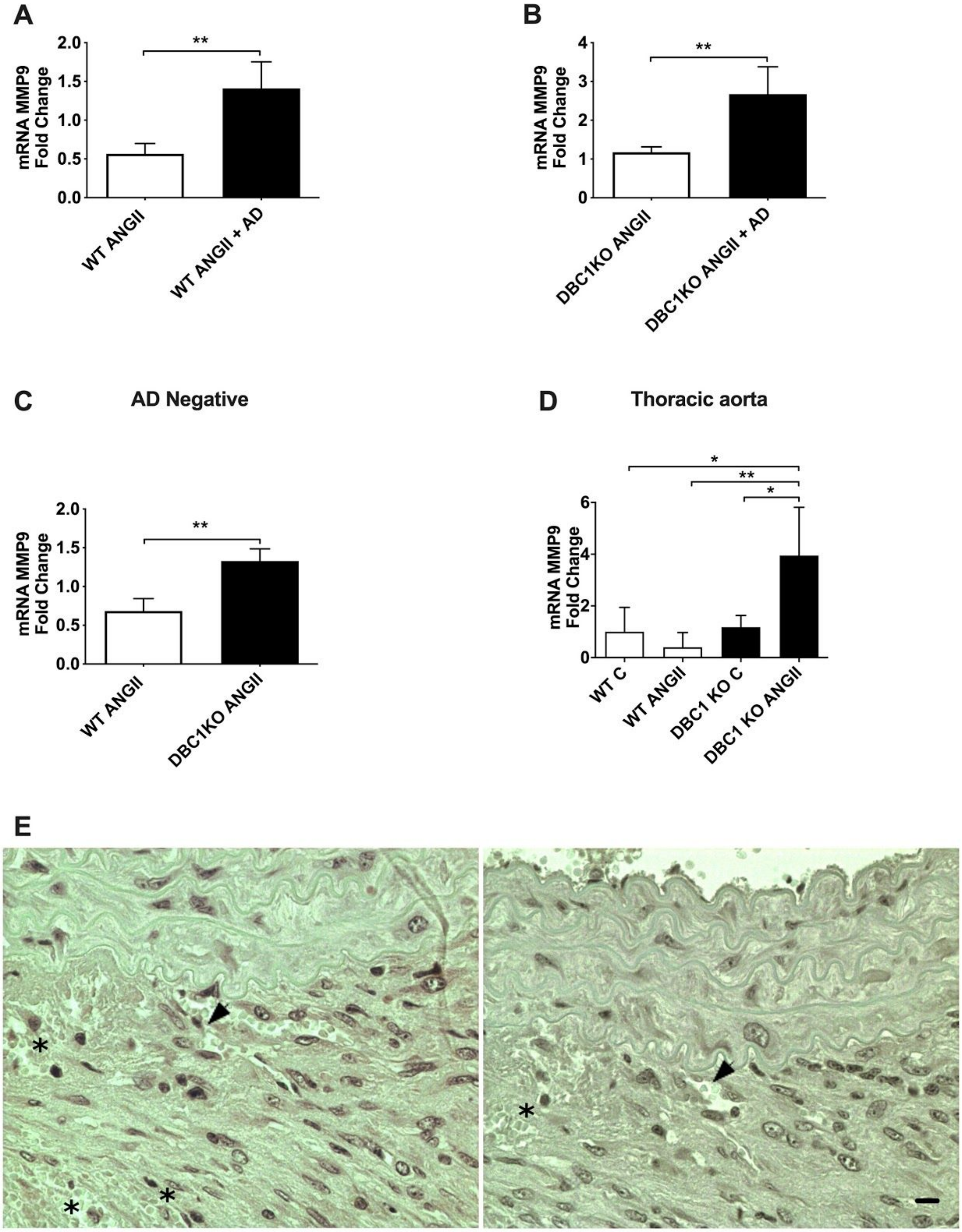
A
mRNA MMP9 Fold Change
2.0
1.5
1.0
0.5
0.0
**
WT ANGII
WT ANGII + AD
B
mRNA MMP9 Fold Change
4
3
2
1
0
**
DBC1KO ANGII
DBC1KO ANGII + AD
C
AD Negative
mRNA MMP9 Fold Change
2.0
1.5
1.0
0.5
0.0
**
WT ANGII
DBC1KO ANGII
D
Thoracic aorta
mRNA MMP9 Fold Change
6
4
2
0
*
**
*
WT C
WT ANGII
DBC1 KO C
DBC1 KO ANGII
E
*
*
*
*

Supplementary figure 2. A-C) mRNA expression of MMP9 in WT and DBC1 KO mice treated with ANGII that did or did not develop AD. Means are expressed as fold change over WT vehicle group. Two-tailed Unpaired t-test was done.** means $p < 0.01$. **D)** mRNA expression of MMP9 in WT and DBC1 KO mice thoracic aortas after 4 weeks of treatment with ANGII or vehicle (C). Thoracic aorta was dissected avoiding any AD tissue for RNA processing. One-way ANOVA with Bonferroni's post hoc test for multiple comparisons were done. * and ** mean $P < 0.05$ and 0.01, respectively (n= 4 per group). **E)** Representative hematoxylin images of thoracic aorta from DBC1 KO mice after 4 weeks of ANGII treatment. (*) signal extravasated erythrocytes. Arrowheads point to ruptures of the vasa vasorum. Scale bar = 10 micrometers

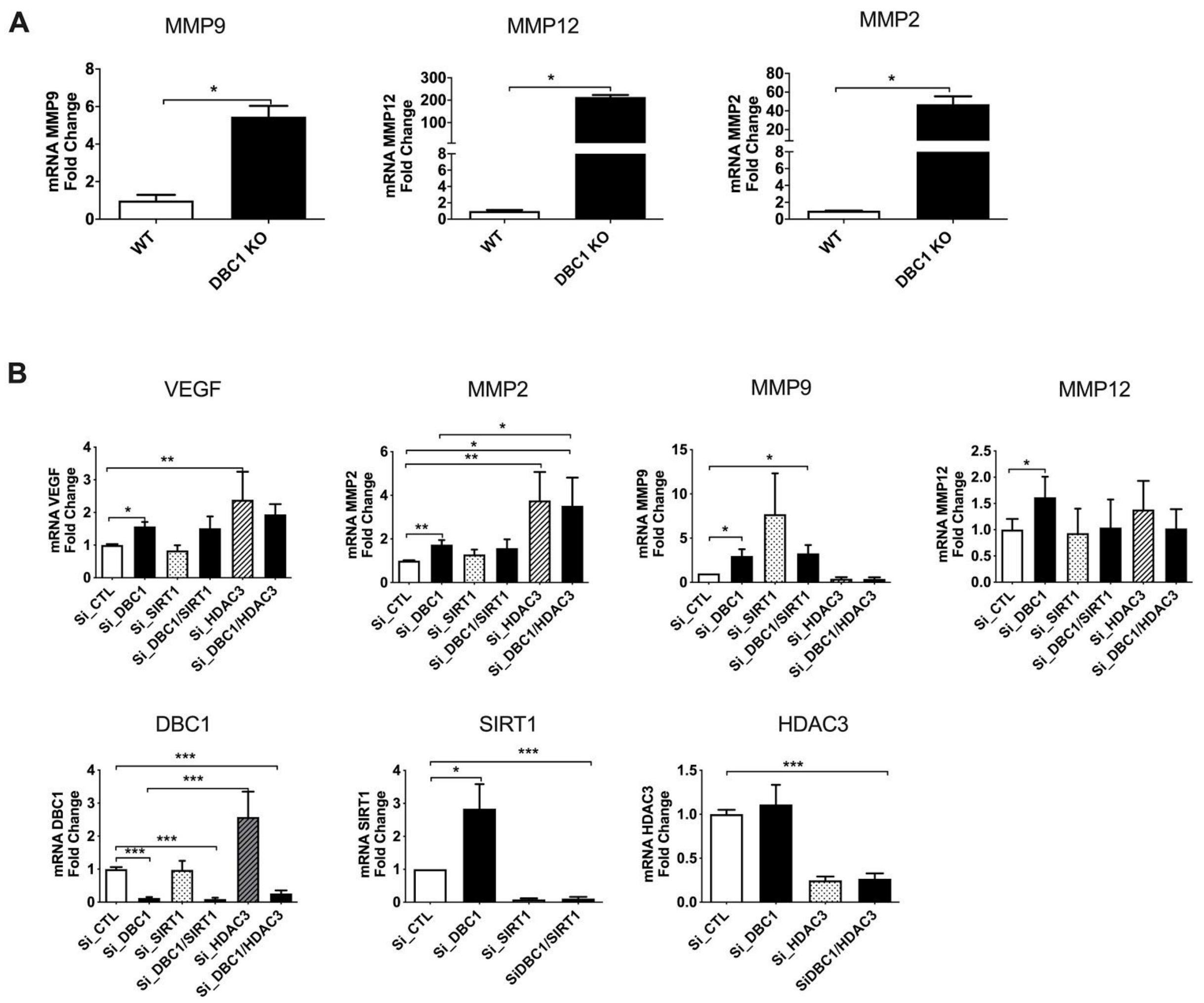

Supplementary figure 3. A) mRNA expression of MMP9, MMP12 and MMP2 in WT and DBC1 KO VSMCs. **B)** mRNA expression of MMP2, MMP9, MMP12, DBC1, SIRT1 and HDAC3 in silenced WT and DBC1 KO VSMCs. The effect of DBC1 on the expression of vascular remodelling factors in VSMC does not depend on SIRT1 or HDAC3. SIRT1 or HDAC3 were knocked-down either alone or in combination with DBC1 in VSMCs isolated from WT mice aortas. The expression of the different vascular remodelling factors was assessed by qPCR 72h after transfection. One-way ANOVA with Bonferroni's post hoc test for multiple comparisons was done (n=4). *, ** and *** mean $p<0.05$, 0.01 and 0.001, respectively.

Supplementary raw data

WB. Figure 3.

Figure. 3B

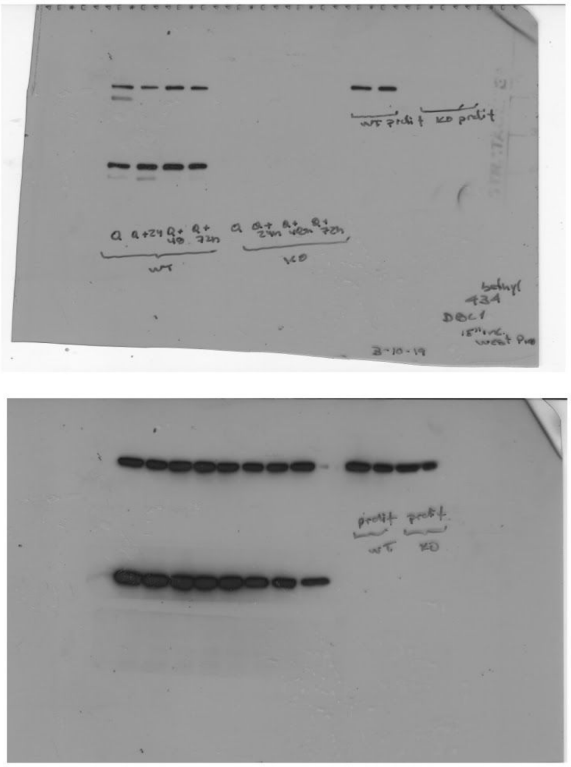

Figure. 3G

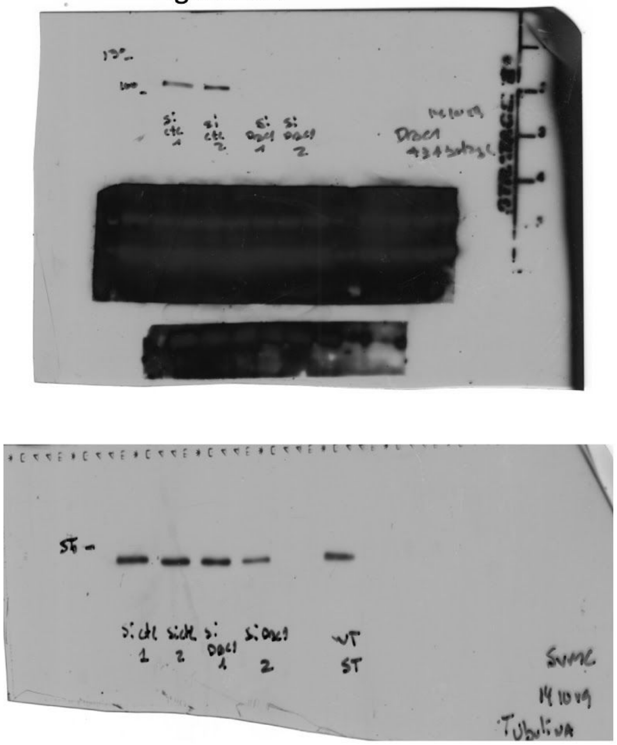

WB. Figure 5.

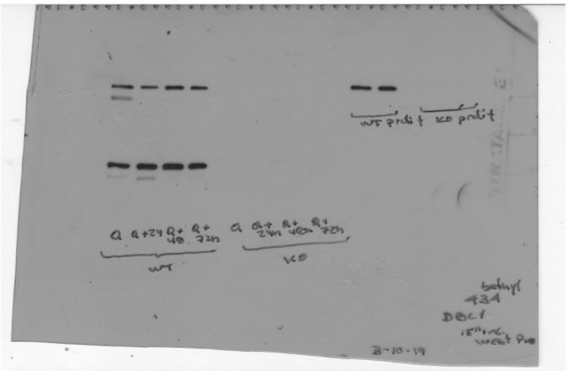

Figure. 5B

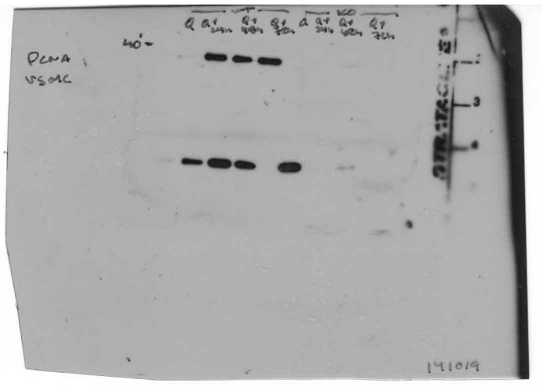

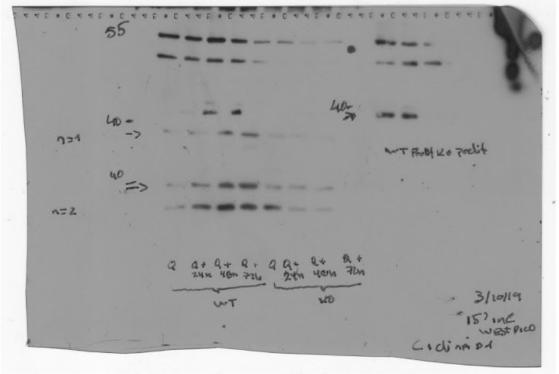

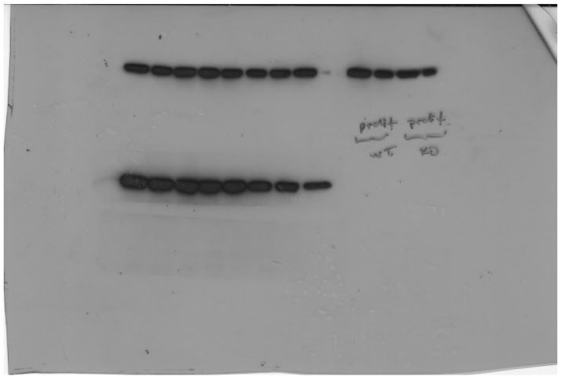

Supplement: Supplementary file 1 — Supplementary Information. [file 41598_2020_63841_MOESM1_ESM.pdf]
